# Uncovering the processes of knowledge transformation: the example of local evidence-informed policy-making in United Kingdom healthcare

**DOI:** 10.1186/s12961-020-00587-9

**Published:** 2020-09-25

**Authors:** John Gabbay, Andrée le May, Catherine Pope, Emer Brangan, Ailsa Cameron, Jonathan H. Klein, Lesley Wye

**Affiliations:** 1 Wessex Institute, University of Southampton, Southampton, SO17 1BJ United Kingdom; 2 Institute of Public Health, University of Cambridge, Cambridge, CB2 0SR United Kingdom; 3 NIHR East of England Applied Research Collaboration, Cambridge, CB2 8AH United Kingdom; 4 School of Health Sciences, University of Southampton, Southampton, SO17 1BJ United Kingdom; 5 Nuffield Department of Primary Care Health Sciences, University of Oxford, Oxford, OX2 6GG United Kingdom; 6 University of the West of England, Bristol, BS16 1QY United Kingdom; 7 School for Policy Studies, University of Bristol, Bristol, BS8 1TZ United Kingdom; 8 Southampton Business School, University of Southampton, Southampton, SO17 1BJ United Kingdom; 9 Bristol Medical School, University of Bristol, Bristol, BS8 2PS United Kingdom

**Keywords:** Evidence-based healthcare policy, research-based evidence, research implementation, knowledge transformation, knowledge mobilisation, healthcare commissioning

## Abstract

**Background:**

Healthcare policy-makers are expected to develop ‘evidence-based’ policies. Yet, studies have consistently shown that, like clinical practitioners, they need to combine many varied kinds of evidence and information derived from divergent sources. Working in the complex environment of healthcare decision-making, they have to rely on forms of (practical, contextual) knowledge quite different from that produced by researchers. It is therefore important to understand how and why they transform research-based evidence into the knowledge they ultimately use.

**Methods:**

We purposively selected four healthcare-commissioning organisations working with external agencies that provided research-based evidence to assist with commissioning; we interviewed a total of 52 people involved in that work. This entailed 92 interviews in total, each lasting 20–60 minutes, including 47 with policy-making commissioners, 36 with staff of external agencies, and 9 with freelance specialists, lay representatives and local-authority professionals. We observed 25 meetings (14 within the commissioning organisations) and reviewed relevant documents. We analysed the data thematically using a constant comparison method with a coding framework and developed structured summaries consisting of 20–50 pages for each case-study site. We iteratively discussed and refined emerging findings, including cross-case analyses, in regular research team meetings with facilitated analysis. Further details of the study and other results have been described elsewhere.

**Results:**

The commissioners’ role was to assess the available care provision options, develop justifiable arguments for the preferred alternatives, and navigate them through a tortuous decision-making system with often-conflicting internal and external opinion. In a multi-transactional environment characterised by interactive, pressurised, under-determined decisions, this required repeated, contested sensemaking through negotiation of many sources of evidence. Commissioners therefore had to subject research-based knowledge to multiple ‘knowledge behaviours’/manipulations as they repeatedly re-interpreted and recrafted the available evidence while carrying out their many roles. Two key ‘incorporative processes’ underpinned these activities, namely contextualisation of evidence and engagement of stakeholders. We describe five Active Channels of Knowledge Transformation – Interpersonal Relationships, People Placement, Product Deployment, Copy, Adapt and Paste, and Governance and Procedure – that provided the organisational spaces and the mechanisms for commissioners to constantly reshape research-based knowledge while incorporating it into the eventual policies that configured local health services.

**Conclusions:**

Our new insights into the ways in which policy-makers and practitioners inevitably transform research-based knowledge, rather than simply translate it, could foster more realistic and productive expectations for the conduct and evaluation of research-informed healthcare provision.

## Background

### Introduction

We have long known that knowledge developed on the ‘high-ground’ of scientific research differs markedly from that used in the ‘swampy lowlands’ of practice [1]. Terms such as ‘practical knowledge’ [2], ‘knowing-in-action’ [1], ‘phronesis’ [3] and ‘knowledge-in-practice-in-context’ [4] have all stressed this categorical difference. Yet, over recent decades, the wave of evidence-based practice and, in its wake, evidence-based policy and management, has created the expectation that healthcare should adhere to the best available scientific, research-based knowledge. How should we resolve this paradox? One way is to establish evidence-based practice on the premise that healthcare professionals cannot implement research-based evidence without inescapably turning it into something different from its original form. If so, this has important practical and epistemological consequences that require an understanding of how and why they do that. Yet, such transformation of knowledge has not been widely studied. We aim here to contribute to that understanding by exploring, within the context of the relevant literature, the findings from a previously published research study [5] in order to examine, in greater depth, the transformation processes carried out by healthcare policy-makers who were using research-based evidence to develop local policies.

### Evidence is transformed when used

The evidence-based healthcare movement has heightened the problem of ensuring that scientifically derived evidence is reliably passed from the researchers who produce it to the policy-makers, managers, clinicians or service users who apply it. Yet, there is a growing recognition that it is naive to envisage this as the simple transfer of knowledge across a research–practice ‘gap’ [6]. Hence, for example, the rise of the term ‘knowledge translation’ and of sophisticated techniques to render scientifically derived knowledge into a form in which it can be used in practice while maintaining maximum fidelity to the research [7–9]. More recent approaches to knowledge translation foster dialogue between researcher and practitioner [10, 11], ranging from simple facilitation, through ‘knowledge brokerage’ and ‘knowledge mobilisation’ [12, 13], to full involvement of the eventual users of the knowledge in its very design and conduct [14–16].

Whilst all these principles and techniques are necessary, they are not sufficient. Davies et al. [14] argue that knowledge uptake goes not only beyond ‘transfer’ but also beyond ‘translation’ thanks to “*the messy engagement of multiple players with diverse sources of knowledge*”. This statement encapsulates two crucial principles. Firstly, the uptake of new evidence is not a linear journey but a multidirectional, multifaceted one – a phenomenon that has been ignored, denied or obscured by over-simplified linear models of knowledge translation [13, 17–22]. Secondly, scientifically derived evidence is only one form of knowledge; the (often tacit) knowledge and experience of care providers and consumers must also be systematically taken into account, along with the demands and constraints of the local context. Healthcare decisions therefore entail not just scientifically derived evidence but also other diverse sources of knowledge [11, 22]. Decision-makers from the many varied backgrounds involved in healthcare differ in how they value those diverse forms of evidence [23], all of which, including scientific research, have their weaknesses as well as their strengths. Thus, the knowledge that ultimately informs decisions normally encompasses far more than the scientific evidence on which it may originally have been based. Davies et al. [14] have consequently declared that terms such as knowledge ‘transfer’ and ‘translation’ “*misrepresent the tasks that they seek to support*” [14] and Greenhalgh and Wieringa [24] have advocated dropping the translation metaphor as it “*constrains thinking*”. Even the term ‘knowledge mobilisation’ now seems inadequate, especially when, as Ward [12] has commented, the literature is “*curiously silent*” about the nature of the knowledge being mobilised. In their observations of multi-sectoral groups formulating local policies about care for older people, Gabbay et al. [25] described in detail their observation of the “*phenomenon of transforming a research finding according to one’s own experience or agenda*”. Based on those case studies and their subsequent ethnographies, Gabbay and le May [4] have portrayed the interactions through which research-based evidence passes during clinical policy-making as “*complex social processes of knowledge transformation*”, during which they likened research-based knowledge to a malleable ball being flipped around a pinball machine, morphing with every impact.

A recent groundswell of evidence from diverse sources supports this conception of knowledge transformation in healthcare, both for clinicians and for managers. May et al. [26] observed that research-based clinical knowledge is “*interpreted and adapted flexibly according to the contingent requirements of specific settings*”. Similarly, Kyratsis et al. [27], in their study of healthcare managers, found that “[e]*vidence was continuously interpreted and (re)constructed by professional identity, organisational role, team membership, audience and organisational goals*”. Nicolini et al. [28] observed that the healthcare chief executives whom they shadowed continually processed and made sense of new knowledge and relied on the skilful and nuanced weaving together of multiple informal and formal sources, which necessarily led to any research-based sources being modified in the process. Croft and Currie, when discussing how healthcare managers acquire, assimilate and transform different types of knowledge into practice, have implied that competing actors reconfigure it in the process [29]. Finally, Swan et al. noted from their case studies that “[k]*nowledge does not travel untouched through social interactions; evidence is modified at the point of decision-making*” [22]. In short, many researchers have found that decision-makers make sense of knowledge by altering it to fit their needs.

There is increasing confirmation that the process of ‘organisational sensemaking’ is usually a collective act; indeed, it has been dubbed “*the social life of information*” [30]. It often involves communities of practice – groups of people who, through chatting about their shared practical interests, chew over and digest new ideas, melding them with their practical, tacit knowledge about the problem and its context to solve problems [31]. Knowledge and professional identity become deeply entwined as they try to arrive at shared (or contested) meanings and negotiated goals and decisions [31, 32].

### The complexities of using evidence

The difficulties of making evidence-based management decisions – let alone policy decisions – are well known [33]. It has long been accepted that managers, like policy-makers, are forced to work with ill-defined goals, resources, problems and options, using whatever is to hand to make hurried, pressurised decisions in ways that are inimical to rational decisions based on solid evidence [34, 35, 36]. Instead, they selectively explore, question and interpret many types of information in light of their existing ideas and the pressures and constraints of their circumstances while seeking justification, often retrospectively, for those judgements [22, 27, 36, 37]. Yet, those responsible for commissioning and managing health services do not find themselves being evaluated for the skill by which they make these intricately negotiated judgements [28, 38], but according to the degree to which they conform to externally delivered guidance and targets based on ‘the (research) evidence’. Moreover, there has been a self-contradictory tendency to insist that healthcare managers, commissioners and policy-makers take maximal account of the views and insights of patients, clinicians and the public, while still adhering as closely as possible to the scientifically derived evidence [28] – logically incompatible aims.

Clinicians too, when making decisions about managing their patients, tend to meld different sources and types of knowledge. In their ethnography of primary care practitioners, Gabbay and le May [4, 39] showed how highly regarded clinicians continually recast many forms of knowledge, including experience and local custom as well as research-based guidelines, into their ‘mindlines’. Mindlines are internalised, collectively reinforced, contextualised, often tacit, informal guidelines for handling complex situations that allow practitioners to navigate the intricacies of practice flexibly but efficiently. Good professional practice relies on taking many, often conflicting, factors into account and rapidly and reliably making sound judgements. Subsequent work has shown that a wide range of clinical professionals use mindlines [40], and yet they too are expected to follow research-based guidance as closely as possible, a demand that risks minimising the necessary practical and contextual judgements that their mindlines afford them.

To summarise, we have argued that there exist strong bodies of evidence that knowledge generated in research is transformed in many ways in the course of its utilisation for healthcare management. The conclusion we draw from the literature cited above is that, while there is understandable pressure on healthcare decision-makers (be they managers or clinicians) to act faithfully according to scientifically derived evidence, the complex milieus in which they operate are inimical to ‘rational’ stepwise decisions based on scientifically derived knowledge. In any given organisational context, research-based evidence undergoes multiple interactions between groups and individuals with differing ideas, needs and values, who may reinterpret or contest research-based evidence, combining it with many other sources and types of evidence. Hence, the knowledge they actually use in practice differs categorically from research-based knowledge. Such a conclusion will come as little surprise to anyone who has tried to implement research in practice.

Yet, we still know remarkably little about the processes by which research-based knowledge becomes practical knowledge. In the belief that better understanding may help to improve the uptake of research-based evidence into policy and practice, our aim in this paper is to explore how, why, where and by whom research-based knowledge is transformed during its uptake. To do so, we use case examples from our published study of policy-makers who have a direct influence on clinical practice, namely National Health Service (NHS) commissioners (Box 1).

### The organisational decision-making of healthcare commissioners

A stream of National Institute for Health Research (NIHR)-funded studies of NHS commissioners has consistently shown their task to be inherently complex and contextually contingent. Checkland et al. [41], for example, described it as “*messy*” and “*fragmented*”, necessitating not just extensive technical skills but also considerable interpersonal interaction. Commissioners had to exchange information in many directions while managing the demands of disparate groups of professionals over whom they had little control [41]. Smith et al. [42] described how commissioners, struggling with a persistent lack of accurate and timely data, would expend extraordinary levels of effort to balance the development of key long-term interpersonal networks with the annual contractual cycle. Swan et al. [15] found that evidence about healthcare did not ‘speak for itself’ – commissioners’ decisions and their evidence base were being co-developed and co-produced in tandem with their own and others’ experiential knowledge and reliant on several ‘inter-dependencies’ stemming from the wide range of processes, tasks and personnel involved. Dopson et al. [43] found commissioners to be engaged in social processes aimed at testing, transposing and contextualising evidence. These studies all confirm that commissioners use evidence by subjecting it to social and interpersonal processes with multiple and complex pathways that go way beyond knowledge transfer or translation.

In this paper, we draw upon our own NIHR-funded study of commissioners. Our focus at the time was the ‘knowledge exchange’ between NHS commissioners and external (mainly commercial) agencies/consultancies [15, 44, 45], but our data also revealed the complex ways in which commissioning organisations processed new knowledge. By examining how they used ‘research-based’ or ‘scientifically derived’ knowledge and evidence (terms that we use here interchangeably) we elucidate the various ways in which commissioners processed it through their organisations en route to embodying it in policy decisions. We explore in depth how, where, why and by whom such knowledge was transformed and examine in detail how the transformations in its nature and meaning resulted from the policy-makers’ interactions with organisational structures and processes.

## Methods

The study had ethical approval from South West Ethics Committee 2 (10/H0206/52). The methods are described in detail elsewhere [5]. They entailed eight case studies between early 2011 and mid-2013, four of which were English commissioning organisations where the commissioners had engaged the help of commercial or not-for-profit agencies; the other four were the external organisations that were providing them with knowledge and expertise. We focus here principally on the four case studies of the commissioning organisations (clinical commissioning groups). The purposive sampling varied the length of time the commissioning organisation had worked with those agencies as well the sites’ geographical and population characteristics and organisational size. LW and EB interviewed 52 people one or more times. This entailed 92 face-to-face or telephone interviews in total, each lasting 20–60 minutes and fully transcribed, 47 (lasting a total of 32.2 hours) of which were with members of commissioning organisations – between 11 and 14 at each site – including chairs, chief executives, board members, clinical leads, commissioning managers, analysts, locality leads, service managers, community representatives and advisers. The other 45 were with interviewees from outside the commissioning organisations: 36 employees of the external agencies, 4 freelance consultants, 3 external public-sector public health professionals, a lay representative and a local authority professional. The interview topic guide in the commissioning organisations included questions about the kind of information that the interviewees felt they needed in order to carry out their commissioning roles, how they accessed and received it, and how it contributed to their decisions. EB and LW observed 14 meetings (41 hours) within the commissioning organisations, including 7 director-level boards, 3 clinical operations committees, 3 unscheduled-care boards and a project board. EB, LW, CP and JHK also observed 11 meetings and training events run or hosted by external agencies, including other NHS organisations as well as commercial consultancies. They documented the activities of each meeting using a topic guide designed to ensure that their notes continually addressed all the research questions. They reviewed 120 documents associated with those meetings, 36 other internal papers, marketing materials, press releases and reports, and 10 websites.

The whole team were involved in analysing the data thematically using a constant comparison method. EB and LW developed an NVivo coding framework iteratively with the team and produced summaries consisting of 20–50 pages for each entire case site, structured around several domains, including models of commissioning, interaction with external providers, and knowledge acquisition and transformation. We also developed agreed ‘thumbnails’ that summarised key findings in each domain. Throughout the study, we frequently and repeatedly discussed and refined emerging findings in informal discussions about the case studies and produced cross-case analyses for all the domains. We also tested the emerging ideas with an advisory group, whose members had practical senior experience of commissioning as well as research. The whole team, including where possible advisory group members, also held 8 regular face-to-face half-day meetings with 14 intervening teleconferences, and a final whole-day facilitated analysis meeting where we critically reviewed all the domains. Here, we draw upon relevant instances from two of the domains – knowledge acquisition and transformation – in all four clinical commissioning group case studies.

## Results

### The use of research-based evidence

Healthcare commissioners (a term we use here to include anyone in the organisation who contributed directly to the work of commissioning health services for the local population) had many roles and came from a wide range of professional backgrounds with clinical, contractual, financial, legal, managerial or epidemiological expertise that helped them to accomplish a wide range of tasks. Some commissioners might be analysts providing internal reports (staff with the technical expertise to, for example, evaluate key performance indicators or crunch patient data, sometimes using commercial software). Some might be redesigning patient pathways or carrying out in-depth analysis on proposed contracts, others might focus on developing business cases to inform the decisions of senior managers, and some might be negotiating contracts with service providers. The art of commissioning was to successfully identify and assess the available options, develop cohesive, compelling arguments for the preferred alternatives and navigate them through the tortuous decision-making system that rendered justifiable decisions acceptable to a range of often-conflicting internal and external opinion. For commissioners to gain the necessary approval they had to negotiate – as our findings will illustrate – competing demands, priorities, hidden agendas, power relationships, professional and personal preferences. Therefore, to a large extent, commissioning entailed repeatedly synthesising appropriate knowledge and information to ensure that it would satisfice (a portmanteau term of satisfy and suffice [46]) the needs of that convoluted – and often financially overstretched – system.

Commissioners acquired information to support their actions and decisions in a wide variety of ways [5, 44]. Sources that were assumed to be supported by the latest research – an assumption the commissioners seldom questioned – included written/online guidance, best-practice summaries, directives, reports, articles, data or surveys. These could be proactively searched or could come directly to them from a very wide variety of national sources, such as the National Institute for Health and Care Excellence (NICE), medical royal colleges, think tanks – mainly the King’s Fund and The Health Foundation – and other professional organisations. Commissioners also sought and/or received evidence-based information indirectly through their professional networks and from co-workers in the form of internal summaries, reports, proposals, presentations or minutes, or more often, through conversations and stories, which were a fast, flexible medium suited to the constantly shifting world of commissioning. Commissioners rarely accessed evidence directly from the scientific literature except where their public health departments or others précised it for them, when it often proved inconclusive, inapplicable or otherwise unhelpful, even when adduced to solve a particular commissioning problem. Given, therefore, that they were usually using multiple pre-digested information sources, there is inevitable uncertainty about the degree to which commissioners were actually processing research-based evidence when arriving at their decisions. In this paper, we will therefore mainly illustrate our argument about the fate of research-based evidence by analysing the way in which commissioners used two key examples of software tools that had ostensibly been developed by incorporating sound research-based clinical and epidemiological evidence into their software. For anonymity, we have named these tools ‘EpiTech’ and ‘PL-Audit’ (Boxes 2 and 3).

### Knowledge behaviours and the negotiation of evidence

Whatever their role, background or task, each actor or group of actors inevitably negotiated each bit of evidence in their own way. (We use the term ‘negotiation’ here in the sense, introduced by Strauss [47], of the implicit and unconscious means of interpreting our world.) Each actor was individually and collectively bringing into play their own expertise, professional norms, values and biases, including their different judgements of the significance of the various types of available evidence. Whether they came across research-based evidence directly or, more often, indirectly through their co-workers, they first had to make sense of it and then navigate it through the system so that they and others could agree a way forward for it. This often entailed weighing it up against local data and experience that might not concur with what the research-based evidence suggested (in which case it was not always the latter that prevailed; local evaluations, even small informal ones, would often be favoured over published evidence-based guidance). Activities that led to such decisions might involve explicit negotiation as well as the implicit negotiation described above. As this occurred, usually in the form of conversations, the substance of the evidence, including its research content, was being handled and interpreted in ways unique to each actor’s particular role and the perspectives it created.

How did the actors behave with the knowledge? Exactly what was involved depended partly on the actors’ often-divergent aims and conflicting pressures. So, for instance, the ‘EpiTech’ software (Box 2) was designed to use research evidence to inform decisions, but each actor or set of actors repeatedly reshaped what they took to be the evidence that EpiTech was giving them. The university-based enterprise supplying the software tool needed to ensure high evidential standards (e.g. clarifying, validating, filtering and adapting the research-based knowledge that the tool was based upon) and to provide robust training (e.g. ‘explaining’ it in understandable terms, ‘simplifying’ it to help potential users adopt it). They promulgated the tool via a local consultancy whose concern was ‘refocussing’ and ‘reorienting’ it to render it easily accessible for use in project management or informatics, and ‘reformulating’ and ‘glossing’ it to produce an attractive user interface. In contrast, the commissioners’ analysts were expected to be ‘probing’ the outputs and their evidential basis, diving deep into the detail, ‘evaluating’, ‘reappraising’ and ‘interpreting’ the information to fully understand the policy implications. The decision-makers needed just the headline results, ‘tweaking’ and further ‘summarising’ the findings to support policies and contracts that they had to devise under the pressures of limited resources and time. Meanwhile the clinicians who were being encouraged to use the tool were ‘challenging’ whether the science-based information it produced reflected real practice; they were ‘contesting’ the EpiTech evidence in the light of other empirical and experiential evidence about their service and, in the main, ‘ignoring’ it. This example was one of many that revealed scientifically derived evidence being submitted to a myriad of knowledge behaviours [25] (in quotation marks) that defied any naive model of its simple translation or mobilisation into healthcare decisions. Whether scientifically robust knowledge had, as here, been acquired via software or via national guidelines, best-practice guidance or local expertise, the actors who were processing and using it transformed it in many different ways when faced with contradictory pressures and sources of evidence. Even when an outside observer might have regarded any initial knowledge source as relatively robust and well-grounded, the actors renegotiated and reformulated the evidence in their own ways so as to help them resolve the challenges presented by their particular segment of the policy-making process.

### Active channels of knowledge transformation

The multiplicity of such transformations of scientifically based knowledge, often resulting from a series of informal conversations between key actors, presented a challenge to discern exactly where, how and why they were happening. However, we were able to identify five dynamically active organisational spaces that fostered activities leading to the transformations – we have termed these ‘active channels of knowledge transformation’ (ACKTs). These constituted the activity areas within which the commissioners continually reshaped the evidence they were working with. We describe the five ACKTs here separately for the sake of clarity but, although distinct, they are inevitably intertwined and interlinked.

**‘Interpersonal Relationships’**, whereby actors exchanged knowledge with colleagues or associates, appeared the most basic and influential ACKT, being also implicitly nested within most of the other channels. Manifesting itself in different ways, this channel most often depended on informal, face-to-face encounters. In one case study, for example, a general practitioner (GP) commissioner noted that sitting close to the Director of Finance and the Director of Public Health in their open plan office had led to a high level of mutual influence. Elsewhere, a public health consultant described how through building personal relationships she had transformed commissioners’ understanding of epidemiologically grounded needs assessment. A freelance analyst working with another commissioning organisation stressed the importance of informal encounters with local GPs, which enabled her to demonstrate personally that she too belonged to the ‘NHS family’, shared their values and allowed them to appreciate each other’s pressures and constraints:

“*If you’ve got trust and if you’ve got experts in both domains really closely engaged, literally, and by that I mean literally sat at the same computer fiddling around with stuff on the screen, bouncing ideas off each other, that for me was where all these light bulb moments came*.” (Freelance analyst)

Where key relationships were poor, outside experts fared less well, especially where they were assumed not to share ‘public sector values’. Recognising this, the (commercial) EpiTech consultants deployed local (NHS) ‘super-users’ to promulgate the commissioning tool rather than doing it directly themselves, so that the personnel working directly alongside GPs did share such values. However, even the super-users, being non-doctors, could not fully share the (powerful) GPs’ professional aims, values and experience, and mostly failed to convert the GPs’ perceptions of EpiTech as a (pointless) ‘clinical management tool’ into the (informative) scientifically derived ‘commissioning tool’ that the commissioning organisation believed it to be.

**‘People Placement’** was an ACKT that capitalised on the active channel of interpersonal relationships, in which the external personnel were deliberately placed long-term among commissioners – ideally working within the commissioning organisation. Examples included a commercial agency co-locating two nurses in hospitals to check commissioning organisation invoices against patient notes, local commissioning support units embedding analytic staff within their client commissioning organisations, and senior managers being fielded at board meetings. Commonly, the intention behind the People Placement ACKT was to inject knowledge into (and also learn about) the healthcare system. Such placements only worked if commissioning organisations were receptive and if care was taken to choose the right person to embed – not only considering the person’s discipline, expertise, technical skills and previous experience in the care sector but also the right soft skills.

“*We were very visible, and we were in the same building as the four commissioning-group leads, directors … very available … So they regularly came to us with questions that weren’t necessarily related to the piece of work that we were doing with them at the time. …. Because we had built that relationship, they didn’t feel threatened*.” (Commercial consultant)

The recurrent dialogues between the embedded consultants and the local commissioners inevitably led to both parties seeing things differently, shaping each other’s perceptions of the information being adduced, including the research-based evidence. Elsewhere, placing NHS staff from different organisations had a similar effect. For example, the local reviewers in the second PL-Audit (Box 3) came from the acute, community, social services and commissioning organisations. Working together to use the data – and hence the research-based evidence – conveyed by the software tool, they pooled and shared their clinical, service and organisational-based knowledge through daily, face-to-face debriefings. This gave the auditors increased power in the ensuing discussions and created new understandings within their respective organisations. So, they were able to successfully bring new insights and policies based on major reinterpretations of the evidence, which also lessened the mutual mistrust between the organisations.

**‘Product Deployment’** was an ACKT that usually entailed the commissioners using commercially developed software tools, e.g. for invoice validation, scenario modelling, audit (e.g. PL-Audit) and, most commonly, risk prediction (e.g. EpiTech) that were grounded in research findings. There were also non-electronic tools, such as a business methods package in line with ‘managerial best practice’. Usually, the commercial provider contributed the method/product and the client brought additional local knowledge. Training (which had patchy success) was part of such Product Deployment, e.g. by webinars to teach clients advanced operational skills for the risk-prediction tool, by holding tutorials for local trainers, or by face-to-face interactive demonstrations of the tool populated with local data. Sometimes, this was very basic training that had little impact. However, in one commissioning organisation, the NHS analysts were the equal of the sophisticated commercial providers and, working closely with them to run and test a scenario-generating tool, made several improvements that the commercial provider incorporated into the next version of the software rolled out to other clients. This was an example of genuine knowledge exchange (and implicit knowledge transformation) whereby both the consultants and their NHS client benefited from changes in the way the tool was used; however, they continued to differ as to the implications of research-based evidence embedded in it and to interpret the evidence in their differing ways.

**‘Copy, Adapt and Paste’** was an important and commonly used ACKT whereby commissioners imported, e.g. patient pathways, service re-designs, tools or ideas from other organisations after subjecting them to (often hard-negotiated) local customisation. It was encouraged by the copious documentation on ‘best practice’ promulgated by the Department of Health, the King’s Fund and other agencies. A commissioning manager described her organisation as constantly ‘horizon scanning’ to look out for pioneering initiatives to appropriate ‘because there are loads out there’. The ‘Copy, Adapt and Paste’ AKCT was so ubiquitous in the case sites that it was sometimes hard to trace the initial sources of innovations, few of which were genuinely original. Moreover, each time an innovation was appropriated into a new context, its content was further modified. For example, after the second PL-Audit (Box 3), once the hospital staff had ‘copied, adapted and pasted’ the audit tool, the lasting knowledge legacy in their future audits was the underlying concept but not the tool itself, thereby entrenching their own clinical knowledge and discarding the United States-derived research-based knowledge enshrined in the PL-Audit. Commissioners in another case study, hearing that a neighbouring county had successfully redesigned patient pathways using a customised commercial desk-top tool based on existing guidelines, tailored it further to meet their own requirements. Its final incarnation looked very different from the original in the neighbouring district.

**‘Governance and Procedure’** was the fifth ACKT that we identified. Unlike the others, whereby commissioners actively sought information, through this channel, they often became passive recipients of information that they were obliged to consider, however sceptically. They were required to take note of, for example, national ‘must dos’ and guidance from statutory bodies but also to consider local activity data and local medical input. However, they regained some control over national requirements during their engagement with that local input, when tussles would emerge within this ACKT over whether or not to implement the guidance, as happened in one site where telehealth, a major national initiative, was opposed by influential local clinicians.

Commissioners’ work was shaped by their statutory role as publicly accountable organisations with prescribed internal structures and procedures. Probity obliged proposals to go through several governance-and-procedure cycles before ultimate approval. The ensuing repeated discussion across several groups made it challenging to determine exactly who took what decisions, when, and based on what formulation of the available information (including research-based knowledge). For instance, we observed a series of meetings where a commissioning board and its sub-committees were required to consider the operational and strategic implications of the Francis Inquiry Report (which provided guidance on improving hospital care nationally following a scandal about poor care.) An ex-NHS freelance consultant synthesised the nearly 300 recommendations in the report into a few categories against which the trust’s Board could benchmark local performance. That simplification proved critically influential in the way the commissioning organisation interpreted the lengthy report’s recommendations and to their subsequent mandatory reviews of ‘measuring up to Francis’.

This ACKT impacted in other ways. For example, when the hospital (Box 3) had proven that the first PL-Audit was misleading despite being based on research evidence, the commissioners were unwilling to rescind the decisions they had based on the audit since the relevant statutory governance committees had already approved them. In other words, the power struggle over the evidence first led to its incorporation into commissioning decisions, then to agreement that it misrepresented the trust’s activity and then, within the Governance and Procedures ACKT, to its retention despite its failings.

What all these examples have in common is that governance and procedural requirements affected the way information, including research-based knowledge, was reconstructed at different stages of the process.

### Incorporative processes

The transformational activities within the ACKTs were underpinned by two crucial and omnipresent incorporative processes – contextualisation and engagement.

‘Contextualisation’, which entailed blending research-based guidance with other sources such as local data, professional and patient experience, or organisational demands and constraints, was about filtering knowledge and focussing it through a local lens. It overcame a common hurdle summarised by one commissioning manager as: “*Someone always says our system is not like that*”. Another told us:

“*I think that’s the crux of our job. It’s really interesting, because you read what you read, and you find out what you can, but then it has to be applied locally. And all localities are different, you know. … And so you have to then balance best practice against what’s reality locally...*” (Commissioning manager)

Views varied:

“*Karen: If evidence or trials show that it works elsewhere then we have to believe that it can work here too. Carol: Agreed, but we can’t assume that it will all work here because the data elsewhere may say 200 but it won’t be 200 here. …*” [The Board later agreed to discuss said evidence with local stakeholders before proceeding.] (Field-notes, commissioning board)

Contextualisation repeatedly appeared in the Product Deployment ACKT, as the knowledge and assumptions built into software tools were often based on scientifically derived evidence or expert consensus from abroad, which needed ‘Anglicising’. Without contextualisation, whether by the external agency or the commissioners themselves, decision-makers tended to dismiss such tools – as happened in PL-Audit (Box 3), where the application of the embedded evidence needed not only major reconfiguration for the United Kingdom context but also specifically for the local hospital (which triggered the Copy, Adapt and Paste ACKT). Even then, commissioners needed advice on applying the local data outputs to their decisions; ideally, they needed access to known and trusted ‘interpreters’ (using the Interpersonal Relationships ACKT). In one commissioning organisation, an external analyst regularly attended meetings (People Placement ACKT) to present a dashboard and work through the local implications of the data with committee members. However, elsewhere, a commissioner worried that without a trustworthy interpreter the wrong conclusions could be drawn:

“*But, you know, what do you do with that data? We know that it must be saying something to us, the fact that a little old lady has had three falls ... But it doesn’t tell us that she’s somebody that necessarily needs to be assessed by the team. … We’ve now got a waiting list for people to be assessed, and half of them, I suspect, will be assessed and it will be decided that they hadn’t really got a big problem at all*.” (GP commissioner)

Contextualisation was central to the Copy, Adapt and Paste ACKT but also manifested itself through the Interpersonal Relationships and People Placement ACKTs, where through relationship-based mingling of knowledge and expertise, research-based evidence could be transformed into a more readily acceptable form. Training on decision-support tools alone did not suffice. The outputs had to be interpreted and contextualised by those who understood the tools (often the ‘analysts’) and passed on to the ‘decision-makers’ who could digest and apply local information, usually after a further round of contextualisation that could include demographic, clinical, political, organisational or financial considerations. When applying national and regional mandates to local circumstances, contextualisation within the Governance and Procedure ACKT entitled local stakeholders to object; this could result in modification or rejection of the mandates, regardless of their basis in scientifically derived evidence.

‘Engagement’ meant promulgating and refining knowledge by informing, training or enthusing those who might either have important information or perspectives or be well positioned to instigate behavioural change (or show good reason not to) and/or might tap into local or national networks to help the initiative succeed. Naturally, the more those individuals were engaged, and the more powerful they were, the more the knowledge was subjected to the behaviours and negotiations discussed above. The Governance and Procedure ACKT was rich in engagement, not least because it required stakeholders to be explicitly consulted and to accept responsibility for decisions. This was often given visibly high priority, but took many different forms with varying success; which stakeholders became engaged and when, and how much power and influence they wielded – or not – could greatly alter the degree and direction of the transformation of the research-based knowledge. Engagement was essential to the successful uptake of the scientifically derived evidence conveyed by the second round of PL-Audit work (Box 3) following the ‘disastrous’ impact of the first audit where there had been no local clinical involvement. (Conversely, the intensive joint working required by clinical engagement helped more generally to remedy the fraught relationships between the hospital and its commissioning organisation.) However, clinical engagement could also thwart naive attempts at evidence-based policy-making; in another case study, hostile clinicians within the commissioning organisation initially disparaged the evidence in national guidance supporting the introduction of telehealth. A compromise policy, buttressed by alternative research-based evidence that backed the clinicians’ scepticism, was eventually agreed. The irony was that, in the face of the two sets of contradictory research-based evidence, the compromise was based on relatively weak local data.

## Discussion

### Summary of findings

Our case studies have amply confirmed, as reported in the literature described in our introduction, that commissioning took place in a multi-transactional milieu resulting in interactive, pressurised, underdetermined and rapid decisions – an environment not of contemplative and logical appraisal of research-based evidence but of continual contested sensemaking through negotiation that involved many other sources of evidence. Our work has enabled us to provide a finer description of the dynamics of this environment and its impact on knowledge utilisation. All types of evidence, including research-based evidence, were repeatedly (re)transformed as commissioners inevitably and incessantly re-interpreted and recrafted it while carrying out their many roles. At every stage, the analysts, managers, consultants, clinicians and others who contributed to commissioning decisions (analysing data, writing reports, making presentations, chairing meetings, meeting deadlines, garnering resources, persuading opponents, finding allies) had their particular motivations, demands and constraints arising from their various specific roles and activities. Therefore, each actor (or group of actors) had to find their own solutions for the best ways to handle such pressures and tensions. The resultant negotiated iteration of the evidence depended on their context-based needs, their experience and tacit knowledge, and their individual and/or collective power.

**Figure 1:**
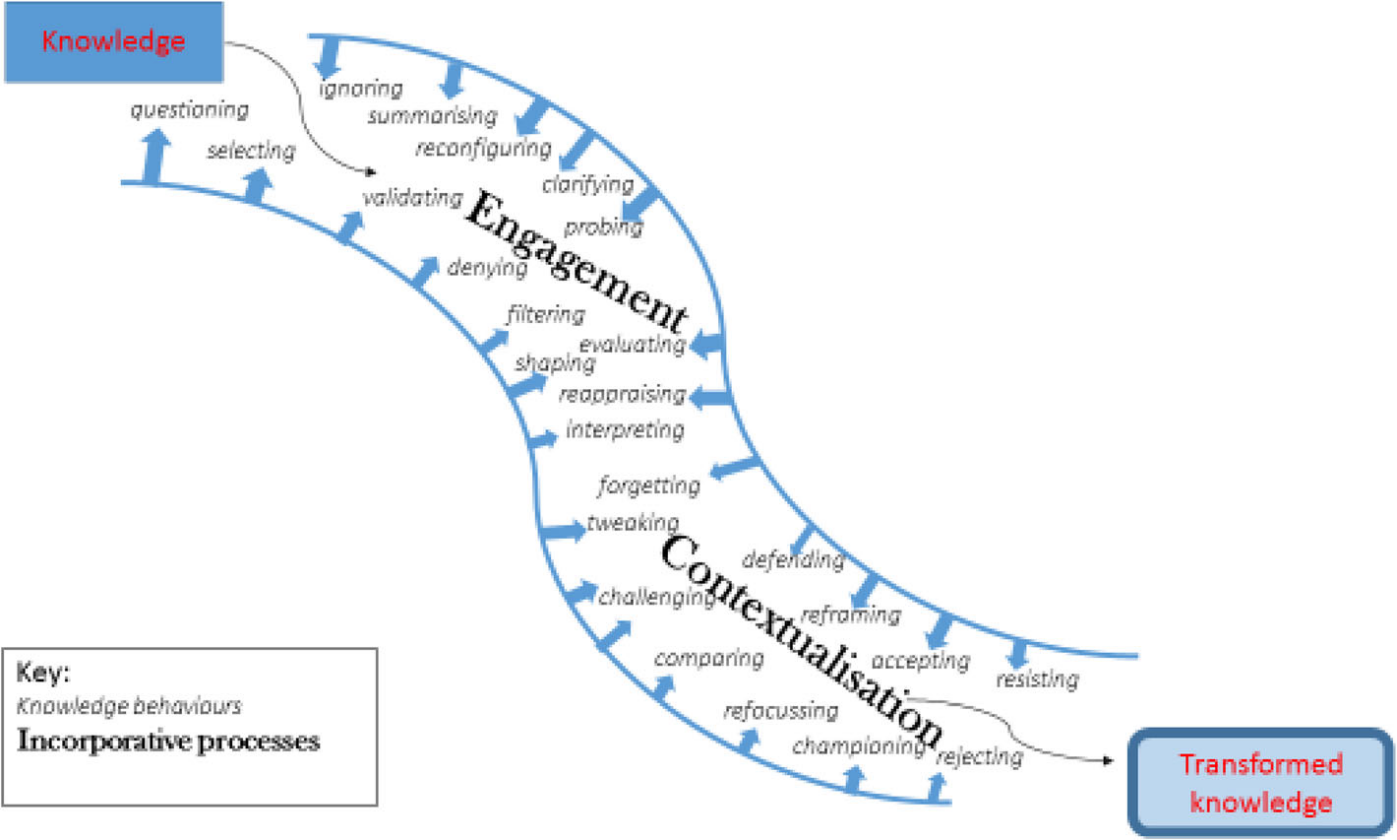
**Fig. 1** What happens in an active channel of knowledge transformation

In analysing where and how that process occurs, we have postulated ‘active channels of knowledge transformation’ that provided both the organisational spaces and the mechanisms for it to happen, fundamentally affecting how and why the evidence was processed, and by whom. Working within those ACKTs, commissioners repeatedly subjected knowledge, including research-based knowledge, to numerous behaviours (questioning it, reconfiguring it, summarising it, etc.), as they negotiated and melded it with other, often conflicting, sources and types of knowledge, beliefs and evidence. This enabled them to maintain two key ‘incorporative processes’, contextualisation and engagement, by which the knowledge was transformed as it was absorbed into the eventual clinical policies that shaped local health and social services (Fig. 1).

### Strengths and limitations

We were able as a deliberately reflexive, multidisciplinary team to draw upon a number of theoretical perspectives and compare across multiple data sources (interviews, observations and documentation) to develop a rich picture of the ways in which managers used research-based evidence. We were also able to iteratively test our emerging ideas with commissioners on our advisory group.

A possible limitation was that the original focus of our study was on the way commissioners exchanged knowledge with external agencies. Therefore, although that aim resulted in a great deal of data about how they subsequently processed that knowledge – on which we have drawn here – our methods were not designed to explore all the factors. This limits our capacity to analyse in any detail, for example, the power dynamics of the ways in which different actors were able to treat and negotiate knowledge or the differences in the way that they took up different types and presentations of research-based evidence. Crucially, we were rarely able to follow a given item of evidence, e.g. a particular best-practice patient pathway, through its various mutations. That would have required extensive shadowing (which the commissioning organisations declined) coupled with detailed discourse analysis, which was beyond the scope of the study.

Another potential weakness may have been the timing of the study, which was disrupted by the 2012 Health and Social Care Act that restructured the NHS, changing the commissioning arrangements. However, we have no evidence to suggest that those changes materially affected the generic structures and processes we report here. Moreover, several of the authors have been in positions that allow us to maintain contact with a range of NHS commissioning organisations, and those contacts give no cause to suppose that the key elements of our findings have altered since our fieldwork.

Finally, we recognise that the five ACKTs that we have identified in these case studies constitute are a beginning to the exploration of knowledge transformation processes rather than the last word. Other organisations will undoubtedly have different types of ACKTs.

## Conclusions

Our general findings correspond to those of previous studies [14, 22] emphasising that NHS clinical policy-makers were working in a turbulent environment redolent of those described in a wide range of management contexts, where simplistic, linear-rational expectations of evidence-based management are unrealistic [33]. We have shown in some detail how, in the messy ‘drama’ of policy-making [48], the actors treated and negotiated research-based evidence to meet conflicting needs [49] through what has elsewhere been called “*pluralism and opportunism*” [50], “*practical rationality*” [51] and “*tailored adaptation*” [52]. Beyond this, we have added empirical detail of how and why the actors responded to the demands and constraints of that context by transforming research-based knowledge into something they could use for the specific task that confronted them at any given stage of what Weiss [17] has called the “*decision accretion*”.

A series of recent studies by a group based at the University of Warwick has also strongly pointed in this direction. They found commissioning managers using “*creativity*” in their “*workarounds*” because they could only use policy directives or other research-based evidence as “*recipes*” that required “*local ingredients*” and skills to actually be put into practice [38]. Hence, commissioning decisions became “*a collective, pluralistic and socially complex endeavour that depends fundamentally on processes of co-production*”, reliant on many “*interdependencies*” that may occur at several stages of an interactive “*evidence journey*” [15].

“*Evidence use is not, then, a one-off event. It reflects the priorities present at a particular point in time. It is entirely plausible that different evidences will have differing utility and weight across episodes of innovation work. For example, [randomised controlled trials] may be used as evidence when identifying solutions to a problem, whereas financial data may be used to understand feasibility. Certain types of evidence relevant in one episode may also be questioned or disregarded in another*.” [22]

Our detailed results confirm and reinforce this suggestion. Our commissioners similarly co-produced their policy deliberations and decisions through multiple interactive processes; they mainly did so within organisational spaces that we have delineated as active channels of knowledge transformation. Crucially, however, we found that this led to them continually reshaping the knowledge that underpinned their decisions, often doing so concurrently, not just episodically as suggested in the quotation above. Each (inter)action resulted in a re-negotiated co-construction – a reformulation – of that knowledge.

Each actor or group of actors has their own set of goals – often at variance with others’ – in trying to solve a particular commissioning problem and move it to the next stage of the process. The pressures and demands on a clinical adviser, for example, would be different from those on a public health analyst or contract manager or indeed subtly divergent from another clinical adviser. In addition to their technical and professional knowledge and skills, each actor has what Swan et al. [22] refer to as “*situated expertise*” based on the varying experience, local knowledge, “*workarounds*” and so on that they have accumulated. One way to frame our findings, then, is to posit that commissioners were using what Gabbay and le May [4] have called the “*contextual adroitness*” that arises from the internalised guidance – the mindlines – that they had collectively and individually accumulated. This would have allowed them to flex their distinctive professional knowledge, skills and techniques, their varied organisational norms, expectations and experiences, their alternative additional evidence, assumptions and values, and enabled them to efficiently negotiate complex decisions. Thus, for any piece of knowledge at any given moment in the process, each actor could formulate their particular rendition of the knowledge-in-practice-in-context necessary to satisfice their contextually distinctive goals and needs [4]. It is this use of their mindlines, we suggest, that enabled the various actors to meld different types of knowledge throughout the commissioning process, allowing them to negotiate their way through the uncertain tensions of contradictory organisational requirements. There was no sudden move from scientifically derived knowledge to applied knowledge but rather a reiterative series of knowledge behaviours and negotiations that constantly altered the form, content and perceived meaning of the research-based evidence underlying policy decisions. Not only does this give the lie to any idea of a (one-off, passive) gap between research and practice, but it also reinforces the fact that one is dealing with (many, active) reconstructions of the scientifically derived evidence. This has profound consequences – although everyone would agree that policy-makers and practitioners should try to abide by ‘the evidence’, it turns out that ‘the evidence’ is disconcertingly elusive. It incessantly changes such that any research-imbued elements that existed may become lost or distorted beyond recognition.

Besides mindlines, one could frame this shifting of the underlying knowledge in other ways. The notion of organisational sensemaking [36], which often takes place within communities of practice [31, 32], is also a highly relevant approach to understanding this phenomenon. The findings described here are consonant with the notion that actors re-create or ‘perform’ knowledge as they engage with it [53]. Outside the realm of healthcare, ideas of performativity have been used to good effect to argue that the reformatting and repurposing of economic theory creates or brings into being the phenomena of economics, in ways that resemble the kinds of performances of knowledge described in our study [54, 55]. Other contributions from the field of Actor Network Theory have similarly shown how different actors within networks actively reconfigure knowledge [56, 57]. Elsewhere, Giddens’ “*dialogical model*” of research utilisation proposes that knowledge is contested, negotiated and emergent [58]. Finally, one can approach this question from the epistemological perspective of the shift from “*propositional knowledge*” to the complex “*narrative knowledge*” that Tsoukas has described in organisational knowledge management [59].

Whatever theoretical frame one chooses, the important point is that it may indeed be worth examining more closely how policy-makers and practitioners interactively transform the nature and content of the evidence itself, and what that means for the conduct and evaluation of research-informed policy-making. Ethnographic techniques such as shadowing will help to unpick exactly how the evidence mutates through the repeated interactions and to explore and test the influence of the active channels of knowledge transformation. These and other methods may help elicit the social psychology of organisational decision-making, not just among policy-makers but also operational managers and clinicians. This will inevitably lead to some difficult epistemological questions about the nature of the different forms of knowledge, including scientifically derived knowledge, that inform policy and practice. However, it is not simply a question of ‘further research needed’. The practical implications of taking seriously the phenomenon of knowledge transformation are paramount.

The processes we have described can have very different outcomes; they may end up badly corrupting research-based evidence, pragmatically adapting it or contextually enhancing it. Once one recognises and accepts that those possibilities always exist, it will become easier to develop ways to discern and realistically monitor the balance between these possible outcomes. Moreover, by understanding the processes by which knowledge is necessarily transformed, one might, rather than naively trying to insist on fidelity, foster the art of discerning when an adaptation maintains the essence of the research findings in a contextually appropriate way, and when it has become so attenuated or distorted as to no longer reflect them. Moreover, rather than trying forlornly to supress the transformation processes, one might be better placed to nurture them so as to augment their benefits, iron out their dysfunctional aspects and minimise their harmful effects. For example, by identifying and enhancing the workings of key communities of practice, or indeed artificially creating them, one might ensure that they have the appropriate membership, shared goals, mutual trust and respect to allow a constructive discussion of the various types of evidence. (This is, for example, what enabled the recipients of the second PL-Audit to accept the evidence that they had previously rejected.) Linked to this is the possibility of facilitating more “*respectful critical conversations*” [60], which was only patchily displayed in our case studies. This may well require the deliberate provision of better “organisational spaces ”[61] for ACKTs to function in, and the need for a deeper understanding of the inherent power relations, so as to enable better dialogue between the various actors [29]. Deeper insights into such matters will also help researchers and knowledge brokers to become more involved than they currently do at many more of the crucial stages of knowledge uptake in complex organisations and to influence the inherent transformations appropriately. A related implication for researchers and guideline producers is that it is unrealistic to expect a one-off encounter, be it an evidence review, a single piece of guidance, or a meeting, to result in the research-based evidence (or any other kind of information) making any noticeable difference.

Recognising and describing the inevitable phenomenon of transformation may also help us evaluate the impact of research on practice. Nearly 40 years ago, Weiss established that policy-makers can rarely point to research that directly informs their decisions since, as she put it, knowledge creeps into their deliberations as decisions gradually accrete [62]. However, it as we have now seen, knowledge does more than creep – it morphs. Thus, if we are to properly discern the place of research-based knowledge in the final policy decisions we need to do more to understand how it gets changed and why. This may allow us to more realistically evaluate the impact of the research, which in turn may help develop more practically relevant research. It may also enable policy-makers and practitioners to face realistic expectations, receive helpful guidance and be fairly evaluated about their use of research-based evidence.

Box 1 The commissioning context at the time of our study (2012–14) In the United Kingdom, local healthcare policy-makers, known as NHS commissioners, are responsible for planning, contracting and evaluating their local healthcare. Their role is to optimise health outcomes by using the best available information to assess local needs, decide priorities and strategies, and then negotiate contracts for the appropriate hospital and community services. From 2012, this function in England was executed by clinical commissioning groups (CCGs) working in a complex environment with senior policy-makers, clinicians and local communities. During our study, there were over 200 local CCGs, each employing general practitioners (GPs) working part-time alongside non-clinical commissioners. CCGs often relied on support from public sector Commissioning Support Units and local government public health departments contributing, for example, evidence reviews, help with analyses of service activity data, project management or contract negotiation. Some CCGs also engaged commercial and not-for-profit providers of software tools and consultancy. At a national level, the National Institute for Health and Care Excellence (NICE) was a key source of guidance about the effectiveness or otherwise of treatments, and an ‘NHS Improving Quality’ initiative offered advice, guidance and tools. NHS England and the Department of Health provided strategic direction by issuing frequent guidance and directives. These and other support agencies, such as think-tanks and health-professional organisations, provided much of the research-based evidence that CCGs were expected to incorporate into their decisions. However, commissioners were also expected to fit these recommendations into their local contexts through analysis of local data and widespread consultation with local health professionals, patients, politicians and public.

Box 2 How EpiTech’s evidence was promulgated to clinical commissioning groups and clinical staff ‘EpiTech’ was a small international company originally from a university in the United States, marketing a software tool, developed through decades of international academic research, that enabled the use of GP practice-level data to generate information about patients with a high likelihood of using health services to a substantial degree. To provide the software, training and the user interfaces, EpiTech relied on local branches of large consultancy companies, whose advantage was their United Kingdom local knowledge. However, the United Kingdom consultancies were selective about the knowledge that filtered through to commissioners, e.g. an informatics company emphasised the delivery of useable patient data, whereas a management consultancy prioritised its managerial consequences, both of which affected the use and usefulness of the knowledge that the tool gave the commissioners. In this case study, keen local analysts, commissioning managers and project managers (who were dubbed locally as ‘super-users’) were selected to learn advanced analytical skills via webinars where EpiTech provided technical training originally oriented to American healthcare but with some attempt to contextualise it for the NHS. The super-users’ roles were to encourage GP practice staff to use the software when planning patient care or to design algorithms., e.g. to help commissioners identify the right people to attend drop-in clinics for older people.

Box 3 How the evidence from PL-Audit was contested and used PL-Audit had used international standards, developed from evidence-based expert consensus on ‘best place of care’, to create a tool with which auditors could review patients’ notes to assess whether they ‘qualified’ to be in their current setting (i.e. hospital versus community care). The commissioners engaged commercial analysts to use the PL-Audit tool in an acute trust to identify hospital admissions that the research-based evidence and international expert consensus groups would have deemed unnecessary. The results suggested that 28% of patients need not have been in hospital, a finding hotly contested by the hospital and placing further strain on their already difficult relations with the commissioners. Over the next 9 months, after the serious shortcomings of the first audit were agreed and its methods revised, the commercial consultants were now to be simply kept on hand in an advisory capacity. In their place, five reviewers from the local hospital, community provider and commissioning agency were trained to use the tool but encouraged to continue augmenting and consolidating the audit methods through the experience of conducting the audit. This involved daily discussions with senior clinicians. The second audit concluded that the proportion of patients ‘not qualifying’ was 24%. Although similar to the earlier, rejected, results, these findings were accepted and readily acted upon within the hospital. The commissioners and hospital trust did not use the tool again but adopted its underlying methods (without the built-in evidence base) in their future audits elsewhere in the hospital.

## Data Availability

The datasets generated and/or analysed during the current study are not publicly available due to commercial confidentiality but are available from the corresponding author (LW) on reasonable request.

## Abbreviations

ACKT: Active Channel of Knowledge Transformation
GP: General Practitioner
NHS: National Health Service
NIHR: National Institute for Health Research
